# Prevalence of and factors associated with burnout among health care professionals in Arab countries: a systematic review

**DOI:** 10.1186/s12913-017-2319-8

**Published:** 2017-07-17

**Authors:** I. Elbarazi, T. Loney, S. Yousef, A. Elias

**Affiliations:** 1 Institute of Public Health, College of Medicine and Health Sciences, United Arab Emirates University, Al Ain, PO Box 17666, United Arab Emirates; 2 Department of Psychiatry and Behavioral Science, College of Medicine and Health Sciences, United Arab Emirates University, Al Ain, PO Box 17666, United Arab Emirates

**Keywords:** Arab World, Compassion Fatigue, Depersonalization, Emotional Exhaustion, Health Personal, Maslach Burnout Inventory, Occupational Health, Personal Accomplishment, Professional Burnout, Wellbeing

## Abstract

**Background:**

Burnout among healthcare professionals is one of the key challenges affecting health care practice and quality of care. This systematic review aims to (1) estimate the prevalence of burnout among health care professionals (HCP) in Arab countries; and (2) explore individual and work-related factors associated with burnout in this population.

**Methods:**

Multiple electronic databases were searched for studies published in English or Arabic from January 1980 to November 2014 assessing burnout (using the Maslach Burnout Inventory; MBI) amongst health care professionals (HCP) in Arab countries.

**Results:**

Nineteen studies (*N* = 4108; 49.3% females) conducted on HCP in Bahrain, Egypt, Jordan, Lebanon, Palestine, Saudi Arabia and Yemen were included in this review. There was a wide range of prevalence estimates for the three MBI subscales, high Emotional Exhaustion (20.0–81.0%), high Depersonalization (9.2–80.0%), and low Personal Accomplishment (13.3–85.8%). Gender, nationality, service duration, working hours, and shift patterns were all significantly associated with burnout.

**Conclusions:**

Within the constraints of the study and the range of quality papers available, our review revealed moderate-to-high estimates of self-reported burnout among HCP in Arab countries that are similar to prevalence estimates in non-Arabic speaking westernized developed countries. In order to develop culturally appropriate interventions, further research using longitudinal designs is needed to confirm the risk factors for burnout in specific HCP settings and specialties in Arab countries.

**Electronic supplementary material:**

The online version of this article (doi:10.1186/s12913-017-2319-8) contains supplementary material, which is available to authorized users.

## Background

Burnout syndrome is a state of physical, emotional, and mental exhaustion that results from prolonged exposure to job stressors or work situations that are emotionally demanding [1–3]. Burnout can have numerous detrimental ramifications on organizations and individuals, such as increased staff turnover, absenteeism, sickness, injury and accidents, low productivity, and interpersonal and organizational conflicts [4]. Health care professionals (HCP) are at a high risk of developing burnout due to the inherent demands and stress of patient care, long and unsociable shift patterns, and an overall high stress environment. Burnout among HCP is one of the emerging challenges affecting health care systems, patient care, and patient safety around the world. Health care professionals may develop symptoms such as anxiety, irritability, mood swings, insomnia, depression, and a sense of failure as a consequence of burnout [5–9]. These symptoms may ultimately lead to decreased job performance and poor patient care [1, 7]. There have been a number of review studies exploring the prevalence and determinants of burnout in HCP in developed and/or westernized countries from North America, Europe, and Australasia [10–15]. However, there is a dearth of reviews focusing on burnout in HCP in developing and non-western countries in the Arab world.

The Arab world consists of 22 countries that are members of the Arab League distributed over two geographical regions Asia and Africa which include Algeria, Bahrain, Comoros, Djibouti, Egypt, Iraq, Jordan, Kuwait, Lebanon, Libya, Mauritania, Morocco, Oman, Palestine territories (Gaza strip and West Bank), Qatar, Saudi Arabia, Somalia, Sudan, Syria, Tunisia, United Arab Emirates, and Yemen [16]. Although some of the Gulf countries are considered high-income countries, all 22 Arab countries are classified as developing countries based on international indicators. The prevalence of burnout may be higher among HCP in Arab countries as their health systems and financing models are either weak, overburdened, or rapidly developing and responding to the changing disease patterns and health status of the population [17]. Many of these countries have a critical shortage of HCP, particularly in some specialties, which may lead to overloading them with work responsibilities and making them prone to burnout. Countries like Iraq, the Palestine territories, Syria, and Yemen have experienced serious deterioration in health care equity and service provision, and also health status due to recent or ongoing civil conflict. The aim of this systematic review was to estimate the prevalence of burnout and explore the factors associated with burnout amongst HCP in Arab countries.

## Methods

This paper has been written in accordance with the Preferred Reporting Items for Systematic reviews and Meta-Analyses (PRISMA) guidelines [18].

### Study design

A systematic review was conducted on studies of burnout prevalence among HCP in Arab countries according to the PRISMA guidelines.

### Eligibility criteria

Studies were included if they: (1) were published in English or Arabic between January 1980 and November 2014; (2) used the Maslach Burnout Inventory (MBI) to measure burnout; and (3) sampled a population of HCP from an Arab country. Studies that did not meet all three criteria were excluded.

### Information sources and literature search

The following electronic databases were searched: PubMed, Science Direct, MEDLINE, Psych INFO, Informit, and ProQuest. References lists of each retrieved paper were searched manually for additional studies. The Public Health in the Arab World (PHAW) database was also searched manually and an email was sent through the PHAW email list to search for any unpublished studies (gray literature) or papers that were not published in indexed journals. Other websites such as the World Health Organization-Regional Office for the Eastern Mediterranean (WHO-EMRO) website, occupational health and mental health websites were also searched manually for possible papers. A manual search by the authors’ name of all retrieved studies to identify additional studies was also conducted. The literature search was last updated on 20 November 2014. A combination of MeSH search terms and keywords were used as follows: ‘burnout’ ‘professional burnout’, ‘health personnel’ or ‘health care professionals’, ‘Arab world’ or ‘Arabic countries’, ‘nurses’, ‘physicians’ and ‘occupational stress’ ‘doctors’, ‘nurses’, ‘allied health personnel’, ‘Middle East’, ‘Arab countries’, ‘Middle East and North Africa region’, ‘Gulf countries’ and ‘Eastern Mediterranean countries’ (see Additional file 1).

### Study selection

Two authors independently screened the titles and abstracts of identified studies and duplicates were removed. Studies considered eligible for full text screening were retrieved for full review.

### Data collection process and data items

Data extracted from each paper satisfying the inclusion criteria was entered into a summary table in the following categories: Authors, Year of Publication, Country, Sample size, Response rate (%), Gender, Professional Group, Individual and Workplace Factors, Prevalence of High EE (%), Prevalence of High DP (%), and Prevalence of Low PA (%). In the current review, prevalence of each dimension of burnout was assessed in each study. No ethical approval was required as the systematic review is based on published data.

### Risk of bias in individual studies

A modified 15-item checklist developed by STrengthening the Reporting of OBservational studies in Epidemiology (STROBE) [19] was used to evaluate the quality of papers reviewed. Three reviewers independently assessed the papers for eligibility and quality, and then met to resolve any disagreements regarding eligibility and/or quality.

### Summary measures

The Maslach Burnout Inventory (MBI) originally developed by Maslach et al. is a standardized tool used by 90% of empirical research to measure burnout [4, 20]. Psychometric analyses conducted on the MBI has shown that the scale has both high reliability and validity (convergent and discriminant) as a measure of burnout [21]. Maslach and colleagues identified three scales that can determine burnout syndrome among professionals that include: Depersonalization (DP), Emotional Exhaustion (EE) and a sense of low Personal Accomplishment (PA) [2]. The MBI instrument contains 22 items that measure the cumulative effects of work-related pressure in three subscales: the EE subscale assesses feelings of being emotionally overextended and exhausted by one’s work (e.g., intense emotional tiredness); the DP subscale measures a negative, cynical, and impersonal attitude towards recipients of one’s service, care, treatment or instruction (e.g., patients); and the PA subscale assesses feelings of competence and successful achievement in one’s work with low personal accomplishment corresponding to demotivation, loss of self-confidence, and self-depreciation in relation to work. Each question is assessed on a scale ranging from 0 (not at all) to 5 (yes, absolutely). A high degree of burnout is represented by high scores of EE (≥27) and DP (≥ 13) and a low score of PA (≤ 31) [2]. As such, the principal summary measure was the prevalence of burnout assessed by the MBI on the three subscales: EE, DP, and PA.

### Risk of bias across studies

The gray literature and proceedings of related conferences were searched to minimize publication bias and articles in both English and Arabic were eligible to minimize language bias.

## Results

**Fig. 1:**
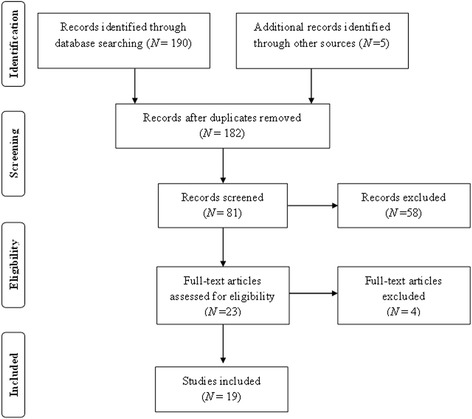
Flow diagram of the search and selection process

The PRISMA flowchart summarizing the data collection process is presented in Fig. 1. We identified a total of 190 articles during the initial search. Ineligible and duplicate studies were removed and 19 papers from eight countries were included in the final review as they satisfied the inclusion criteria. Included studies consisted of seven papers from Saudi Arabia, three papers respectively from Lebanon, Palestine and Egypt, and one paper each from Jordan, Yemen, and Bahrain. The key information from the included studies is summarized in Table 1. All papers except for one were published in indexed journals and the HCP in the reviewed papers included doctors, nurses, physiotherapists, social workers, and occupational therapists.

**Table 1:** Summary of papers and findings by country and year

Authors, Year of Publication	Country	Sample size	Response rate (%)	Gender	Professional Group	Individual and Workplace Factors	Prevalence High EE (%)	Prevalence High DP (%)	Prevalence Low PA (%)
1. Jahrami et al. [42]	Bahrain	153	58.0	Males (48.0%), females (52.0%)	Doctors (9.2%), nurses (78.4%), other healthcare professionals (12.4%)	Job satisfaction	24.2	18.3	28.1
2. Mohammed et al. [28]	Egypt	84	64.6	Males (54.8%), females (45.2%)	Residents	Working hours Burnout was correlated with different personality domains and working hours	81.0	64.3	52.4
3. Shams and El-Masry [26]	Egypt	98	73.1	Males (73.5%), females (26.5%)	Anesthesiologists: Residents (30.6%), assistant lecturers (32.7%), lecturers (16.3%), assistant professors (12.2%), professors (8.2%)	Job stress, working condition, lack of support	62.2	56.1	58.2
4. Khashaba et al. [36]	Egypt	140	100.0	All males	Emergency medical responders	Organizational stressors	20.0	9.3	19.3
5. Hamaideh [24]	Jordan	181	82.3	Males (55.8%) and females (44.2%)	Nurses	Job satisfaction	54.7	34.2	38.7
6. Ashkar et al.[30]	Lebanon	155	75.0	Males (55.5%) and females (44.5%)	Residents (different specialties)	Coping with stress at work, number of on calls	67.7	47.1	37.4
7. Alameddine et al.[35]	Lebanon	755	NR	Males (49.6%) and females (50.3%) 0.1% missing	Doctors (44.8%), nurses (32.7%), allied health in primary health care and other health professionals (22.5%)	High burnout, lower level of education, and low tenure were all associated with increased likelihood to quit	23.2	13.8	18.7
8. Sabbah et al. [25]	Lebanon	200	95.2	Males (31.5%) and females (68.5%)	Nurses	Work satisfaction, work overload, job title, shift duty	77.5	36.0	33.0
9. Abdallah [40]	Palestine	180	NR	Males (33.3%) and females (66.7%)	Social workers	Self esteem	20.0	46.7	13.3
10. Abushaikha and Saca-Hazboun [34]	Palestine	152	59.6	Males (26.3%) and females (73.7%)	Staff nurses (80.3%), head nurses (12.5%), assistant head nurses (7.2%)	Job satisfaction	37.5	9.2	39.5
11. Alhajjar et al.[41]	Palestine	176	70.4	Males (48.3%) and females (51.7%)	Social workers	Employer type	56.2	67.0	85.8
12. Sadat-Ali et al. [39]	Saudi Arabia	69	51.9	NA	Orthopedic surgeons (consultants and specialists)	Hospital type; Government (G) or Private (P)	Overall = 50.7 G = 40.8;P = 70.0	Overall = 59.4 G = 49.0; P = 80.0	Overall = 17.0 G = 14.5; P = 30.0
13. Al-Dardas et al. [23]	Saudi Arabia	198	79.0	NA	Nurses	Nationality, marital status area of work and job duration	45.6	42.0	28.5
14. Al Turki [37]	Saudi Arabia	37	61.6	All Females	Saudi Nurses	Job duration	45.9	48.6	13.6
15. Al-Ayoubi and Jan [22]	Saudi Arabia	130	65.0	Males (45.0%) and females (55.0%)	Pediatricians: consultants, (46.0%), residents (31.0%), assistants (23.0%)	Hospital types, years of practice, number of clinics per week	No subscales prevalence 18% Normal (MBI score 20–30) 82% Abnormal (MBI score >30) a. 19% Mild (MBI score 31–40) b. 29% Moderate (MBI score 41–60) c. 34% Severe (MBI score 61–80)		
16. Aldrees et al. [29]	Saudi Arabia	348	74.0	Males (72.0%) and females (28.0%)	Consultants (54.0%), residents (46.0%)	Number of years in practice, worked concurrently in the private sectors	54.0	35.0	33.0
17. Al-Sareai et al. [38]	Saudi Arabia	370	94.9	Males (81.9%) and females (18.1%)	Primary health care doctors: residents (98.1%), specialists (1.4%), consultants (0.5%)	Number of working days, age and nationality	29.5	15.7	19.7
18. Al-Imam and Al-Sobayel [33]	Saudi Arabia	119	72.4	Males (37.0%) and females (63.0%)	Physiotherapists	Workload, control, reward, fairness, values	42.0	33.6	28.5
19. Al-Dubai and Rampal [27]	Yemen	563	70.4	Males (59.5%) and females (40.5%)	Doctors: specialists (30.0%), non-specialists (70.0%)	Long working hours, work-life balance, dealing with patients, and not chewing Khat	63.2	19.4	33.0

*Note* *DP* denotes Depersonalization, *EE* denotes Emotional Exhaustion, *NR* denotes Not Reported, *PA* denotes Personal Accomplishment

All the included papers were cross-sectional studies with the majority employing a convenient sampling method. The study sample size varied from 37 to 755 with a response rate ranging from 58.0 to 100.0%. A total of 4108 HCP were included in the review. The majority of the studies involved doctors (*N*=2169) from various departments and specialties; 518 primary healthcare and family physicians; 475 medical residents, 90 pediatricians, 68 anesthesiologists, 69 orthopedic surgeons, and the rest were distributed among other specialties. Nurses ranked second with a total number of 1135 from different specialties, followed by social workers (*N* = 356), emergency responders (*N* = 140), physiotherapists (*N* = 119), occupational therapists (*N* = 14), and the occupation of 175 participants was not clearly identified. Almost half (49.3%) of the study samples were females based on the 17 papers that reported the participants’ gender. Out of the 4108 HCP included in the review, approximately one-third (*N* = 1271; 30.9%) were working in Saudi Arabia from various backgrounds. The nationalities of participants were reported in all papers except the study in Bahrain.

The prevalence of each burnout dimension (EE, DP and PA) was reported in 18 papers while one study only reported the overall prevalence of burnout [22]. A high level of burnout among nurses was reported in three studies from Saudi Arabia, Jordan, and Lebanon, [23–25]. Other studies concluded that burnout levels of general practitioners, residents and specialists were higher compared to other health professions [26]. The burnout prevalence for the three domains ranged from 20.0 to 81.0% for emotional exhaustion (EE), 9.2 to 80.0% for depersonalization (DP) and 13.3 to 85.8% for low personal accomplishment (PA). A study in Yemen reported a significant association between higher burnout levels and younger age group of doctors [27]. Similar findings were found in studies conducted in Egypt, Saudi Arabia, and Lebanon [28–30]. Two papers reported a significant association between female gender and EE [23, 26]. Other factors associated with high burnout included being single and nationality [25–27, 31, 32].

Several studies examined the relationship between burnout and workplace factors. The study from Yemen found a significant association between the prevalence of burnout and working long hours. Specifically, doctors working more than 40 h per week had a higher level of burnout (OR = 2.1, 95% Confidence Intervals 1.3–3.6, *p* < 0.01) [27]. Similar findings were reported by a study in Lebanon which found HCP working for more than 80 h per week, and having more than eight on-calls per month were predictive of burnout in medical residents [30]. Years of practice, work duration, work load, type of the organization, shift rotations and duty times were the most commonly reported factors associated with burnout [22, 25, 29]. Low job satisfaction, a poor reward system, and injustice were also associated with a high level of burnout [24, 33, 34].

## Discussion

The primary aim of this study was to estimate the prevalence of burnout among HCP in Arab countries. Our study findings revealed moderate-to-high but wide-ranging prevalence estimates of burnout among HCP in Arab countries. For example, two studies conducted in Lebanon reported high prevalence estimates of EE among doctors (67.7%) [30] and nurses (77.5%) [25]; whereas another study conducted in Lebanon reported a lower prevalence of high EE (23.2%) among doctors and nurses [35]. However, the prevalence of high DP was reported to be 70.7% in one study [35] compared to the other two studies 47.1% [30] and 36.0% [25]. Similarly, two studies [26, 28] conducted on doctors in Egypt reported a high prevalence of high EE (62.2%, 81.0%, respectively), high DP (56.1%, 64.3%, respectively) and low PA (52.4%, 58.2%, respectively) compared to a study on emergency medical responders in Egypt that reported a lower prevalence of high scores across all three sub-scales (20.0% EE, 9.3% DP, 27.0% PA) [36]. Prevalence for high burnout in Saudi Arabia showed similar variability across doctors, nurses, and physiotherapists with EE ranging from 29.5 to 54.0%, DP from 15.7 to 48.6%, and PA from 17.0 to 71.5% [23, 29, 37–39]. In agreement with these findings, the prevalence estimates for all three sub-scales for nurses and social workers in Palestine ranged considerably between the three studies [34, 40, 41]. Overall, studies conducted in Bahrain [42] and Jordan [24] reported some of the lowest prevalence estimates of high EE (24.2%, 32.7%, respectively), DP (18.3%, 27.7%, respectively), and low PA (26.8%, 26.8%, respectively) across all three sub-scales. It is unclear why there were wide-ranging estimates for burnout both within- and between-countries. However, organizational (e.g., organizational climate, management/leadership styles, horizontal and vertical communication) and/or individual factors (e.g., demographic characteristics, personality characteristics, individual attitudes, maladaptive coping styles) that differ across health systems, health care facilities, and HCP within- and between-countries may be responsible. Future work should focus on elucidating the organizational and individual factors that are associated with burnout in HCP working in Arab countries. Finally, there was considerable methodological and statistical heterogeneity between studies and this may account for a portion of the variability in burnout prevalence estimates.

Only one study [23] mentioned whether the study sample was composed of nationals or expatriate HCP and this is particularly important for the Gulf countries, such as Bahrain, Kuwait, Qatar, Saudi Arabia, and the United Arab Emirates that have large multinational expatriate HCP populations. The study found that non-Saudi Arabian nurses were significantly more prone to high EE than Saudi Arabian nurses (27.3% ± 12.1% versus 21.6% ± 2.9%; *p* < 0.01), whereas there were no significant differences between the prevalence estimates of high DP and low PA [23]. Two cross-sectional studies conducted in Kuwait [31] and Qatar [43] were not included in this review as they did not provide the prevalence of burnout (Kuwait) or utilize the standardized MBI to assess burnout (Qatar). The study in Kuwait randomly selected two out of five health regions (Capital and Farwaniya) and sent the MBI questionnaire to all 378 physicians working in primary healthcare and family centers in these areas and 200 physicians (44.0% male; 49.0% Kuwaiti national) completed the MBI (52.9% response rate). Males, non-Kuwaitis, aged more than 40 years, working for more than 10 years, holding only a bachelor degree, with no hobby, and suffering from at least one chronic disease were personal factors significantly related to emotional exhaustion. Non-Kuwaiti national general practitioners working in the Farwaniya area were more likely to suffer from depersonalization. However, higher-income, specialist Kuwaiti physicians that were married and with a hobby, working in the Capital health area were factors associated with higher personal accomplishment [31]. Similarly, a cross-sectional survey was conducted among general practitioners working in 21 primary healthcare centers in Qatar using the Astudillo and Mendinueta questionnaire; scores ranged from 0 to 48; burnout = score ≥19) [43]. One-hundred and eighty-three general practitioners (50.8% male; 20.2% Qatari) responded (79.5% response rate) and 12.6% were classified as burned out with the prevalence of burnout significantly greater among females (28.1%) compared to males (6.9%), and also Qatari nationals (37.8%) compared to expatriates (11.6%) [44]. Both of these studies suggest that there may be a relationship between citizenship, or at least the personal and workplace factors associated with permanent residence, and burnout. Currently, there is a lack of research exploring the factors associated with burnout amongst expatriates versus nationals in Gulf countries; however, perceived stability of employment/residence and isolation from family in their home country may partly explain the higher prevalence of burnout amongst expatriate HCP working in Arab countries. Future research is needed to validate these suggestions.

The category of HCP and the types of departments and specialties were not specified in all papers. However, we were able to categorize the participants from all selected papers into three main types: studies that assessed a mixed group of HCP that worked in a specific institution or department; studies that assessed a specific group of HCP that worked in different departments; and studies that assessed a specific group of HCP who worked in a specific department and a specific specialty. A few studies assessed one category of HCP such as physiotherapists, mental health workers, orthopedic surgeons, anesthesiologists, pediatricians, and non-medical emergency responders while the rest included a mixed group of HCP that included nurses and doctors and other allied health care professionals that were not categorized in some of the studies. Twelve studies concluded that physicians and residents were suffering from higher burnout than reported international prevalence estimates with some differences depending on the specialty and the nationality. There were only three studies included in this review that assessed specialist physicians. Orthopedic surgeons in Saudi Arabia were found to have a similar prevalence of burnout as pediatricians [22]. The same study demonstrated that age, gender, and setting of work were significantly associated with burnout levels. Similar findings were found in a study which reported that 70% of physicians from different specialties had high burnout levels [29]. Higher levels of burnout were found among obstetrics and gynecology, family medicine, anesthesia, intensive care, internal medicine, and cardiology specialties. Moreover, a study conducted among anesthesiologists found that 62.2% of the subjects experienced high EE, 56.1% had high DP, and 58.2% low PA [26]. The high prevalence of burnout amongst these specialties may be due to higher case load and/or complex cases. Residents were categorized in some of the studies and were found to have high levels of burnout with the highest prevalence in the EE domain followed by DP and then low PA [28–30]. The study among residents in Egypt concluded that residents were potentially more prone to higher burnout levels due to the high demands placed upon them, long working hours, insufficient income, a perceived mismatch between effort and reward, and poor hospital administration [28]. Studies exploring the level of burnout among nurses only reported moderate to high prevalence estimates across all three subscales suggesting that this group of HCP may also be at a higher risk of burnout [23–25, 34].

Although most studies focused on doctors and nurses, there were several studies that evaluated burnout among other HCP; including social workers, physiotherapists, occupational therapists, and mental health workers. Two studies assessed burnout levels in a representative sample of social workers in the West Bank and Gaza, Palestine [40, 41]. Both studies showed that social workers had high burnout levels across different sub-domains; however, the overall prevalence of burnout was higher in social workers in Gaza than compared to the West Bank (high EE 56.2 vs. 20.0%, high DP 67.0 vs. 46.7%, low PA 85.8 vs. 53.3%; respectively) [40, 41]. Currently, there is a lack of research focusing on burnout in HCP in the Palestine territories and the difference in burnout prevalence between social workers in Gaza compared to the West Bank may be due to the unique political, social, and cultural conditions of these areas. In other occupations within healthcare settings, moderate levels of high burnout were reported for emergency medical responders in Egypt (EE 20.0%, DP 9.3%, PA 27.0%) [36]. A recent paper in Saudi Arabia also reported a moderate level of burnout among physiotherapists with high EE in 42.0% of participants and 39.4% of participants had a moderate level of DP while 37.8% of the subjects showed low PA [33]. Future burnout research in Arab countries may want to consider including the wider spectrum of HCP including dentists, dieticians, medical technicians and laboratory scientists, midwives, pharmacists, and radiographers.

The prevalence estimates reported in this study are comparable to the prevalence in non-Arabic speaking countries such as Canada, France, United Kingdom, and the United States. Results of burnout in all three dimensions were congruent and in some studies exceeded levels of self-reported burnout in studies conducted in westernized and/or developed countries such as Belgium [44] (hospital nurses 75% high EE), England [45] (mental health nurses 52% high EE, 38% high DP, 60% low PA), Japan [46] (psychiatrists 21% high EE, 12% high DP, 72% low PA), Poland [47] (hospital nurses 71% EE, 40% DP, 77% low PA), Scotland [48] (psychiatric nurses 27% high EE, 7% high DP, 33% low PA), the United States [49], and Wales [50] (community mental health nurses 36% high EE, 12% high DP, 10% low PA). However, self-reported burnout amongst nurses in Australia [50, 51] (mental health psychiatric nurses 36% high EE, 24% high DP, 23% low PA, forensic psychiatric nurses 22% high EE, 17% high DP, 20% low PA [51]; rural psychiatric nurses [52] 10% high EE, 12% high DP, 12% low PA) and Finland [53] (mental health and psychiatric nurses 29% high EE, 21% high DP, 16% low PA), were lower than other westernized developed countries and also compared to the estimates from Arab countries. Interestingly, burnout estimates for hospital nurses working in Iran, a Muslim Persian country, were lower than nurses working in Muslim Arab countries (23% high EE, 5% high DP, 21% low PA) [54]. At present, there is a dearth of research directly comparing the prevalence of burnout between Arab Muslim countries such as in the Arabian Gulf and neighboring Persian Muslim country Iran. The Iranian sample of nurses (*N* = 180) working in public hospitals in Shiraz were all graduates of either technological educational institutions (67%) or universities (33%) and the majority of nurses were married (73.9%) with 0–6 children and a mean (± SD) work experience of 2.8 ± 1.4 years. The Iranian sample may have contained nurses that were older, more educated/trained, and with a greater number of years of nursing experience. These factors may account for the lower levels of burnout in the Iranian nurses.

The second aim of this review was to explore the various personal, interpersonal, and organizational factors that may be associated with burnout in HCP. Among 210 Lebanese nurses who worked in different hospitals and departments, high EE was higher among nurses aged 30–39 years and among married nurses who had higher EE, DP was highest among nurses in the private sector, and low PA was highest among nurses in the public sector [25]. In addition, DP was higher among nurses who worked night and rotating shifts [25]. In Jordan, a study found that 54.7% of mental health nurses (*N* = 181) had a high level of EE [24]. The author also reported that low PA was found to be higher in older Jordanian nurses, those who do not intend to leave their job, and nurses with longer psychiatric experience. The study suggested that the high levels of burnout among these HCP were associated with a poor psychosocial work environment, low social support from colleagues and supervisors, or by working with difficult and uncooperative patients with a poor prognosis. These findings were similar to those of the study in Bahrain which demonstrated that psychiatric HCP in a hospital, majority were nurses, had high burnout levels comparable with other studies that assessed staff in mental health institutions [42]. Most of the studies suggested that general workplace and organizational factors were associated with HCP burnout; however, there was variability in the associations between the proposed factors and burnout. Two studies did not find an association between supervision or lack of control/decision making and burnout [30, 36]; while two other studies reported a negative correlation between low social support from colleagues and supervisors with high burnout among nurses and anesthesiologists [24, 26]. A study conducted among 755 primary health care providers in Lebanon reported that respondents with a high level of burnout on the PA subscale had 3.05 times the odds of quitting their jobs [35]. The study also found that an increase in the number of work hours per week and full-time employment status were associated with reduced likelihood to quit [35]. Burnout among Saudi Arabian physiotherapists was associated with some factors such as years of experience and age, role, and workload [33]. Specifically, there was an inverse relationship between DP and perceived reward and fairness; and a positive relationship between workload and EE [33]. Interestingly, a recent cross-sectional study conducted in Saudi Arabia assessed the prevalence of burnout (October to December 2014) amongst a convenient sample of Saudi Arabian nationals working as critical care nurses (*N* = 150) in three government-run general hospitals in Hail and reported a significant negative relationship between EE and job satisfaction [55]. This finding may have important implications for staff turnover as job satisfaction may strongly influence an individual’s intention to leave a healthcare organization or profession [56]. Future studies exploring the prevalence of burnout in HCP working in Arab countries should endeavor to collect information on job satisfaction and intention to leave using standardized measures. Such data would enable researchers to explore the relationships between burnout, job satisfaction, and intention to leave.

### Limitations

The main study limitations include the low number of studies that have been conducted among HCP in Arab countries and the poor quality and reporting of methodology and results in the majority of papers. As such, intra- and inter-country comparisons of burnout prevalence were limited by methodological differences such as sampling, recruitment, data collection, and analysis. Future studies assessing the prevalence of burnout among HCP in Arabic populations using standardized methodologies are needed. A few studies explored the association between various personal, workplace, and other environmental factors and the three domains of burnout; however, the majority of these studies did not use multivariate techniques and so the results need to be interpreted with caution. Future studies should attempt to collect additional data on factors that may be associated with burnout, such as marital status, years of clinical experience, place of work, location, specialty, shift pattern, perceived support from supervisors and support from co-workers, and self-rated job strain. Our search strategy involved conducting independent searches on six different electronic databases for papers published in English or Arabic between January 1980 and November 2014; and searching the PHAW database and contact list to retrieve unpublished papers or papers not published in indexed journals. Despite these efforts, it is possible that papers meeting our inclusion criteria were missed and therefore, not included in the review.

## Conclusion

In view of the ongoing changes in the economic, social, and political environment in the majority of Arab countries, further studies are needed to identify the factors that are leading to the high level of burnout among HCP in the Arab world. This systematic review reinforces the need to reduce occupational stressors by improving the workplace environment and developing culturally-relevant resilience building programs for HCP working in Arab countries. The burnout literature would benefit from more high-quality studies with larger samples that identify HCP by occupation/specialty and other risk factors related to burnout such as age, personal factors, workplace factors, and other environmental factors. Future studies assessing the prevalence of burnout among HCP in Arab populations may want to consider standardizing the study methodology to compare within- and between-country estimates of burnout prevalence. Further studies are needed to assess burnout among specific HCP and to determine the profile of HCP that are usually at-risk of burnout. Identifying those at risk will help practitioners develop coping and wellness programs that might alleviate some of the factors that lead to stress and burnout in HCP. Future studies using longitudinal designs would allow researchers to explore the causal risk factors and assess whether self-reported burnout fluctuates over time.

This systematic review shows moderate-to-high estimates of self-reported burnout among HCP in Arab countries that are similar to prevalence estimates in non-Arabic speaking westernized developed countries. Specific occupations and specialties appear to be at a higher risk of burnout and the type of work, duration, workload, and other personal factors seem to be associated with burnout level. Many studies did not measure or report the organizational and workplace factors potentially associated with burnout; therefore, future studies need to assess and report all important factors.

## Additional file

Additional file 1: Literature Search Details. Literature Search Syntax for PubMed. (DOCX 23 kb)

## Abbreviations

DP: Depersonalization
EE: Emotional exhaustion
HCP: Healthcare professionals
MBI: Maslach Burnout Inventory
PA: Personal accomplishment
PHAW: Public Health in the Arab World
PRISMA: Preferred Reporting Items for Systematic reviews and Meta-Analyses
STROBE: STrengthening the Reporting of OBservational studies in Epidemiology
WHO-EMRO: World Health Organization-Regional Office for the Eastern Mediterranean
