# Supplementary material for: Prevalence of and factors associated with burnout among health care professionals in Arab countries: a systematic review
Source: BMC Health Serv Res. 2017 Jul 17;17:491. doi: 10.1186/s12913-017-2319-8 (PMC5513024; doi:10.1186/s12913-017-2319-8)
Supplement: Additional file 1: — Literature Search Details. Literature Search Syntax for PubMed. (DOCX 23 kb) [file 12913_2017_2319_MOESM1_ESM.docx]

**Additional file 1:** Example of search syntax and details used for PubMed.

**PubMed Search Syntax**

("professional burnout"[All Fields] OR "burnout"[All Fields] OR "occupational stress"[All Fields]) AND ("health personnel"[All Fields] OR "health care professionals"[All Fields] OR "nurses"[All Fields] OR "physicians"[All Fields] OR "doctors"[All Fields] OR "allied health personnel"[All Fields]) AND ("Arab world"[All Fields] OR "Arabs"[All Fields] OR "Arabic countries"[All Fields] OR "Middle East"[All Fields] OR "Middle East and North Africa region"[All Fields] OR "Gulf"[All Fields] OR "Eastern Mediterranean"[All Fields] OR "Western Asia"[All Fields] OR "Asia"[All Fields] OR "Trucial States"[All Fields])
